# High Variability in Cellular Stoichiometry of Carbon, Nitrogen, and Phosphorus Within Classes of Marine Eukaryotic Phytoplankton Under Sufficient Nutrient Conditions

**DOI:** 10.3389/fmicb.2018.00543

**Published:** 2018-03-27

**Authors:** Nathan S. Garcia, Julie Sexton, Tracey Riggins, Jeff Brown, Michael W. Lomas, Adam C. Martiny

**Affiliations:** ^1^Department of Earth System Science, University of California, Irvine, Irvine, CA, United States; ^2^Bigelow Laboratory for Ocean Sciences, National Center for Marine Algae and Microbiota, East Boothbay, ME, United States; ^3^Department of Ecology and Evolutionary Biology, University of California, Irvine, Irvine, CA, United States

**Keywords:** eukaryote, protist, diatom, dinoflagellate, prymnesiophyte, temperature, cell size, growth

## Abstract

Current hypotheses suggest that cellular elemental stoichiometry of marine eukaryotic phytoplankton such as the ratios of cellular carbon:nitrogen:phosphorus (C:N:P) vary between phylogenetic groups. To investigate how phylogenetic structure, cell volume, growth rate, and temperature interact to affect the cellular elemental stoichiometry of marine eukaryotic phytoplankton, we examined the C:N:P composition in 30 isolates across 7 classes of marine phytoplankton that were grown with a sufficient supply of nutrients and nitrate as the nitrogen source. The isolates covered a wide range in cell volume (5 orders of magnitude), growth rate (<0.01–0.9 d^−1^), and habitat temperature (2–24°C). Our analysis indicates that C:N:P is highly variable, with statistical model residuals accounting for over half of the total variance and no relationship between phylogeny and elemental stoichiometry. Furthermore, our data indicated that variability in C:P, N:P, and C:N within Bacillariophyceae (diatoms) was as high as that among all of the isolates that we examined. In addition, a linear statistical model identified a positive relationship between diatom cell volume and C:P and N:P. Among all of the isolates that we examined, the statistical model identified temperature as a significant factor, consistent with the temperature-dependent translation efficiency model, but temperature only explained 5% of the total statistical model variance. While some of our results support data from previous field studies, the high variability of elemental ratios within Bacillariophyceae contradicts previous work that suggests that this cosmopolitan group of microalgae has consistently low C:P and N:P ratios in comparison with other groups.

## Introduction

The average ratio of elements in marine plankton has traditionally been thought to center on the Redfield ratio at 106 moles of carbon (C):16 moles of nitrogen (N):1 mole of phosphorus (P) (Redfield, 1958). However, the stoichiometry of elements within marine organic particles is variable between biogeographical provinces (Martiny et al., 2013; DeVries and Deutsch, 2014; Teng et al., 2014), and within phytoplankton isolates (Geider and La Roche, 2002), which suggests that the average oceanic C:N:P is plastic, and perhaps changes over time as a function of interacting physical and biological factors. Environmental factors like light, temperature, and nutrients influence phytoplankton physiology and cellular elemental content, potentially molding relationships between phytoplankton phylogeny and cellular elemental stoichiometry (Rhee, 1978; Laws and Bannister, 1980; Urabe et al., 2002; Finkel et al., 2006; Toseland et al., 2013; Garcia et al., 2016; Lopez et al., 2016). In order to understand how factors contribute to regional differences in elemental stoichiometry and field microbial populations, analyses need to separate physical and biological factors.

Systematic relationships between phylogeny and cellular elemental stoichiometry have been linked to the evolutionary and environmental history of major phytoplankton lineages (Ho et al., 2003; Quigg et al., 2003). For example, Quigg et al. (2003) suggests that algae with green plastids have higher C:P and N:P ratios than other groups with red plastids, which may be related to the evolution of ocean chemistry. Aside from this relationship, field studies have identified differences in C:P and N:P ratios between two lineages of cold-water phytoplankton with red plastids near Antarctica (Arrigo et al., 1999, 2002) that are as large as differences observed between laboratory cultures of phytoplankton with green and red plastids (Quigg et al., 2003); where *Phaeocystis* (Prymnesiophyceae) has high C:P and N:P ratios in comparison with diatoms (Bacillariophyceae). Although previous laboratory studies (Quigg et al., 2003) focus on high growth rate conditions to minimize potential effects of variable physiology on elemental stoichiometry, physiological variability may be very different between major phytoplankton groups in the natural environment. Linking physiological variability with variability in elemental stoichiometry between taxonomic groups in field studies may be key to identifying how taxonomic shifts in phytoplankton communities might influence biogeochemical cycles within large biogeographical provinces. Thus, determining the relationship between phylogenetic structure, environmental growth conditions and cellular elemental stoichiometry is key to understanding how phytoplankton interact with biogeochemical cycles through time (Deutsch and Weber, 2012).

To identify systematic relationships between environmental gradients and cellular elemental stoichiometry, analyses need to separate phylogenetically correlated traits from other effects (Finkel et al., 2005, 2006, 2007, 2010; Mouginot et al., 2015). For example, small phytoplankton belonging to marine Cyanobacteria may have high C:P and N:P ratios relative to eukaryotic lineages with larger cells (Bertilsson et al., 2003; Martiny et al., 2013). However, laboratory data indicate that eukaryotes can also have high C:P and N:P ratios (Goldman et al., 1979). To gain a more in-depth understanding of how phylogenetic structure is related to cellular elemental stoichiometry of marine eukaryotic phytoplankton, we analyzed the relationship between cellular C:N:P ratios and the 18S ribosomal RNA sequence of marine eukaryotic phytoplankton isolates. We asked the question: Does phylogeny structure relationships between cellular elemental stoichiometry and gradients like cell size, growth rate, and temperature? Our isolate selection includes wide ranges in phylogeny, cell volume, and temperature habitats from which phytoplankton cells were originally isolated. With respect to variability in cellular elemental stoichiometry, our data suggest that deep phylogenetic structure may not be as important as other factors that influence cellular elemental stoichiometry of marine eukaryotic phytoplankton, such as environmental controls on physiology and other biological factors.

## Methods

We measured the elemental composition of 30 isolates from the National Center for Marine Algae and Microbiota (NCMA) culture collection representing 7 classes within the kingdoms Chromista and Plantae. For taxonomic nomenclature and hierarchical organization, we utilized the World Register of Marine Species (www.marinespecies.org). Cultures within the class Bacillariophyceae were grown in L1 medium (mole N:mole P = 24.4) and others were grown in L1 medium without silicate. We grew isolate cultures at temperatures that were close to the ambient ocean temperature from which isolates were originally collected, yielding 4 groups based on temperature, ranging between 2 and 24°C. Not all cultures were axenic although we used stringent culturing methods to prevent contamination. Cultures were maintained at temperatures very close to the ambient temperatures from which they were collected. Light was supplied with daylight white fluorescent lamps between 50 and 80 μmol quanta m^−2^ s^−1^ on a 13 h light:11 h dark incubation cycle. We monitored cultures daily with *in vivo* fluorescence of Chl *a* (Figure S1) and growth rates were calculated over 2-day periods and plotted in Figure S2. Cultures were terminally harvested for the analysis of cellular elemental composition, cell size measurements, and 18S rRNA sequence analysis approximated 1–2 weeks after they were initiated. For the analysis of growth rate, we used fluorescence data from the last 2 days before cultures were terminally sampled. This time frame did not necessarily align with the maximum observed growth rate (Figure S2). All samples were collected 3–4 h after the beginning of the photoperiod. Triplicate samples for the analysis of carbon and nitrogen (20–25 ml) and phosphorus (20–25 ml) were collected onto precombusted GF/F filters (450°C, 4 h) under low pressure vacuum filtration. Samples for DNA extraction were collected either by filtering 10–20 ml onto a polycarbonate filter or by pelleting cells with a centrifuge. Particulate organic carbon and nitrogen were analyzed with an elemental analyzer (Flash EA 1112 NC soil analyzer, Thermo Scientific) with acetanilide as a standard. Particulate organic phosphorus was analyzed with a spectrophotometer using methods described in Garcia et al. (2016).

For 17 isolates, we used 18S rRNA sequence data from the National Center for Biotechnology Information database and we sequenced this region from the remaining cultures. DNA was extracted using a DNA extraction kit (D6001; Zymo, Irvine, CA). Primers for PCR were prepared by Integrated DNA Technologies, Inc. (Coralville, IA) and selected based on eukaryotic 18S rRNA sequence data as provided by Wang et al. (2014), which amplified the region between the ~300 and ~1500th position on the 18S rRNA sequence. Primer sequences are: forward−5′CGGAGAGGGAGCMTGAG3′; reverse−5′GCATCACAGACCTGTTATTGCC3′, and had a melting point between 56.0–56.4°C. The sequences of the PCR products were determined with Sanger sequencing by Laragen, Inc. (Culver City, CA). The consensus sequence for the 30 isolates was determined with Geneious 9.0.4 (Biomatters, Inc., Newark, NJ) and aligned with the SINA aligner (Pruesse et al., 2012) provided by Silva (www.arb-silva.de). We built a phylogenetic tree of the 30 isolates using Phylip 3.695 (Felsenstein, 1989; 100 bootstrap, F84 distance model, and neighbor joining with *Schizosaccharomyces pombe* as an outgroup). The phylogenetic position of each lineage matched past phylogenetic analyses.

To broadly compare the phylogeny of our cultures with their cellular elemental ratios, we used the Mantel test (from the “vegan” package in “R”; Oksanen et al., 2015), to compare distance matrices of the 18S rRNA sequence computed as above and of elemental ratios compared as a Euclidean matrix. To further separate phylogenetic relationships between the 18S rRNA sequence and cellular elemental stoichiometry with other factors including growth rate, temperature, and cell size, we used the comparative analysis of phylogenetic relationships with a phylogenetic generalized linear model from the “caper” package in “R,” with lambda set at maximum likelihood and kappa and delta fixed at 1.0 (Orme, 2013).

## Results

We used two statistical tools (i.e., phylogenetic least squares regression model and the Mantel test to compare distance matrices) to examine how phylogeny and physiology affect cellular elemental stoichiometry within broad and narrow ranges of phylogenetic groups of marine eukaryotic phytoplankton. First we examined general statistical characteristics such as means and ranges of mole ratios of C:N:P, cell volume and growth rate data across isolates (Table 1). We then quantified how cellular C:N:P within the isolates varied as a function of phylogenetic diversity with a matrix correlation (Mantel test) and as a function of the interaction between phylogeny and physiology with phylogenetically corrected linear statistical models. We designed our isolate selection so that diatoms covered a large fraction of the selection in order to determine how factors like cell size might control cellular elemental stoichiometry within this globally-abundant and biogeochemically-important phytoplankton lineage.

**Table 1:** Molar ratios of cellular elements within isolates of eukaryotic phytoplankton curated at the National Center for Marine Algae and Microbiota.

**Class**	**Species**	**Isolate**	**Temp (°C)**	**μ (d^−1^)**	**s.d**.	**μ_max_ (d^−1^)**	**s.d**.	**μ: μ_max_**	**Cell volume (μm^−3^)**	**s.d**.	**C:N**	**s.d**.	**N:P**	**s.d**.	**C:P**	**s.d**.
Cryptophyceae	*Chroomonas mesostigmatica*	CCMP1168	20	0.31	0.01	0.58	0.04	0.54	150.7	65.3	6.81	0.22	26.81	1.20	182.4	3.9
Dinophyceae	*Heterocapsa neie*	CCMP448	20	0.11	0.02	0.41	0.02	**0.27**	3, 513.3	1, 366.8	7.31	0.10	9.99	0.24	72.9	0.9
	*Amphidinium carterae*	CCMP121	24	0.00	0.03	0.52	0.00	**0.00**	870.2	318.7	7.85	1.74	17.67	4.39	133.7	1.4
	*Prorocentrum mexicanum*	CCMP687	24	0.11	0.01	0.24	0.04	0.46	8, 441.5	2, 611.1	9.44	0.28	10.39	1.27	98.3	15.1
	*Karenia brevis*	**CCMP2281**	24	0.11	0.01	0.33	0.03	0.33	13, 129.0	3, 873.5	7.18	0.11	16.40	1.11	117.8	9.5
Prymnesiophyceae	*Phaeocystis antarctica*	CCMP1374	2	0.06	0.02	0.28	0.05	**0.22**	94.0	55.0	6.08	0.30	18.48	2.66	111.7	10.9
	*Gephyrocapsa oceanica*	**CCMP2051**	20	0.22	0.02	0.53	0.02	0.41	87.1	43.4	7.04	0.14	17.88	2.32	126.1	18.3
	*Gephyrocapsa oceanica*	CCMP2054	20	0.43	0.04	0.52	0.12	**0.83**	142.0	60.0	5.69	0.58	25.57	8.79	142.4	38.2
	*Emiliania huxleyi*	CCMP2090	20	0.36	0.01	0.71	0.03	0.51	390.9	213.8	7.39	0.10	18.17	0.43	134.3	2.8
	*Phaeocystis globosa*	CCMP1528	24	0.83	0.03	0.95	0.03	**0.87**	79.8	4.2	6.37	0.16	16.49	3.94	105.0	24.1
Bacillariophyceae	*Pseudo-nitzschia sp*.	CCMP1309	2	0.12	0.05	0.32	0.02	0.39	200.3	63.6	5.89	0.03	13.90	1.03	81.9	6.5
	*Fragilariopsis cylindrus*	CCMP1102	2	0.14	0.07	0.28	0.08	0.48	38.9	21.1	5.72	0.13	10.10	0.36	57.8	1.7
	*Thalassiosira minima*	CCMP991	2	0.11	0.05	0.40	0.10	**0.27**	790.4	243.9	5.76	0.21	11.75	1.71	67.7	10.0
	*Thalassiosira nordenskioleldii*	CCMP992	2	0.10	0.02	0.28	0.04	0.35	1, 156.2	478.7	6.97	0.12	14.57	0.50	101.6	2.2
	*Thalassiosira rotula*	**CCMP1018**	14	0.12	0.05	0.54	0.01	**0.23**	2, 190.4	1, 206.3	6.11	1.59	29.58	1.13	181.8	54.2
	*Thalasiosira guillardii*	**CCMP988**	14	0.17	0.03	0.58	0.00	**0.29**	117.5	44.8	6.61	0.16	17.33	0.27	114.5	2.4
	*Thalassiosira pseudonana*	CCMP1335	20	0.76	0.01	1.04	0.02	**0.73**	42.4	28.6	8.89	0.08	13.61	0.76	121.0	6.0
	*Thalassiosira weissflogii*	CCMP1587	20	0.56	0.10	0.79	0.09	**0.71**	564.7	154.1	7.22	0.22	19.45	8.11	139.4	53.8
	*Phaeodactylum tricornutum*	**CCMP633**	20	0.45	0.04	0.64	0.04	0.70	154.5	84.2	5.64	0.31	12.83	2.60	71.8	10.6
	*Phaeodactylum tricornutum*	CCMP2561	20	0.69	0.01	0.77	0.01	**0.89**	229.1	366.4	5.23	0.06	15.61	1.21	81.6	5.3
	*Thalassiosira oceanica*	**CCMP1005**	24	0.92	0.04	1.04	0.03	**0.88**	91.5	3.2	4.85	0.18	12.20	3.04	75.9	30.9
	*Thalassiosira weissflogii*	CCMP1050	24	0.46	0.10	0.91	0.02	0.51	734.0	207.4	8.21	0.39	14.36	0.64	117.8	7.2
Dictyophyceae	*Aureococcus anophogefferens*	**CCMP1790**	20	0.30	0.02	0.60	0.05	0.49	14.3	10.2	7.36	0.26	13.33	1.32	98.0	7.3
	*Pelagomonas calceolata*	**CCMP1865**	20	0.20	0.02	0.55	0.03	0.36	13.2	7.3	6.06	0.16	18.06	1.86	109.4	9.8
	*Pelagomonas calceolata*	**CCMP1756**	24	0.34	0.03	0.88	0.03	0.39	10.5	6.3	5.60	0.26	20.35	3.35	113.5	14.4
Mamiellophyceae	*Micromonas pusilla*	**CCMP485**	14	0.22	0.01	0.59	0.06	0.38	5.3	3.0	7.37	0.34	12.38	2.77	90.7	16.9
	*Micromonas pusilla*	**CCMP1723**	20	0.01	0.02	0.54	0.00	**0.02**	5.9	4.2	6.39	0.19	8.89	1.22	56.9	9.0
	*Ostreococcus lucimarinus*	**CCMP2972**	20	0.07	0.02	0.55	0.02	**0.13**	0.1	0.0	5.25	0.17	22.81	2.13	119.5	7.3
	*Micromonas pusilla*	CCMP2709	24	0.00	0.04	0.47	0.10	**0.01**	4.6	3.7	5.63	0.53	12.20	4.56	67.1	18.3
Prasinophyceae	*Pycnococcus provasoli*	**CCMP3400**	20	0.21	0.05	0.51	0.03	0.42	5.9	3.8	7.87	0.30	15.85	1.94	125.1	19.3

*Isolates were grown and curated near field temperatures from which they were collected at 50–80 μmol quanta m^−2^ s^−1^. Growth rate (μ) data were collected during the 2-day period just prior to sampling triplicate cultures for cellular elemental ratios. Observed maximum growth rate data (μmax, dependent on culture conditions) were calculated from the maximum change in relative fluorescence (Figure S1) over a 2-day period and are plotted in Figure S2. The standard deviation (s.d.) is reported. Phylogenetic nomenclature and heirarchical organization is based on information from the World Registry of Marine Species. Bold isolate identification numbers indicate isolates from which DNA was extracted, amplified with 18S rRNA primers and the PCR product sequenced. Other sequence data were collected from the National Center for Biotechnology Information. Mean μ relative to the observed mean μmax was not constant between isolates. Values of mean μ: mean μmax below 0.30 are bolded and highlighted in blue and values above 0.70 are bolded and highlighted in red*.

**Figure 1:**
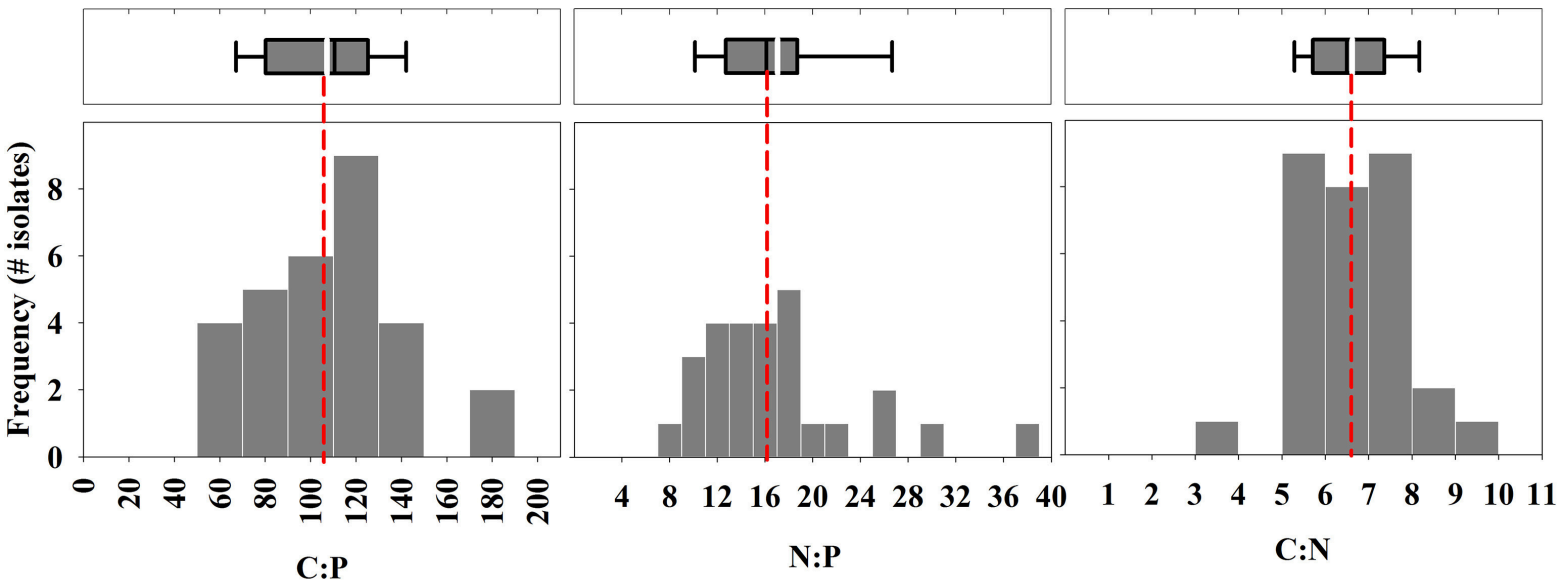
Frequency of isolates within binned intervals of molar elemental ratios of carbon, nitrogen, and phosphorus. Bin intervals for C:P, N:P, and C:N are 20, 2, and 1, respectively. Top box-whisker plots include the mean (white line) and median (black line) of ratios for all 30 isolates. The box indicates quartiles and the dotted line provides a reference to the Redfield ratio of C:N:P (106:16:1).

**Figure 2:**
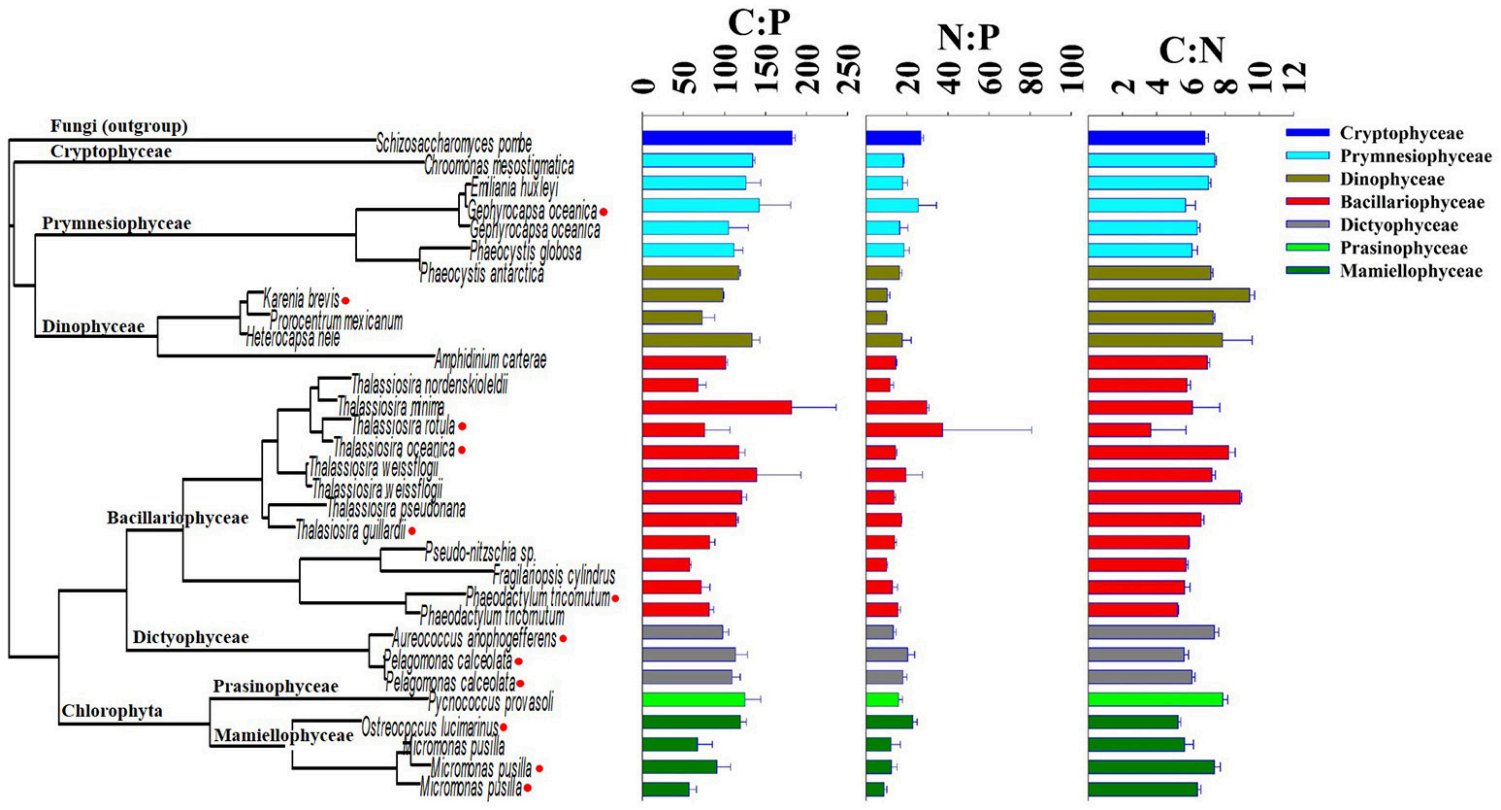
Phylogenetic tree of the 18S rRNA sequence of 30 eukaryotic phytoplankton isolates in comparison with the molar ratio of cellular elements of carbon, nitrogen, and phosphorus. Red markers on the tree indicate isolates that were sequenced by Laragen, Inc., vs. others that were collected from the National Center for Biotechnology Information database.

**Figure 3:**
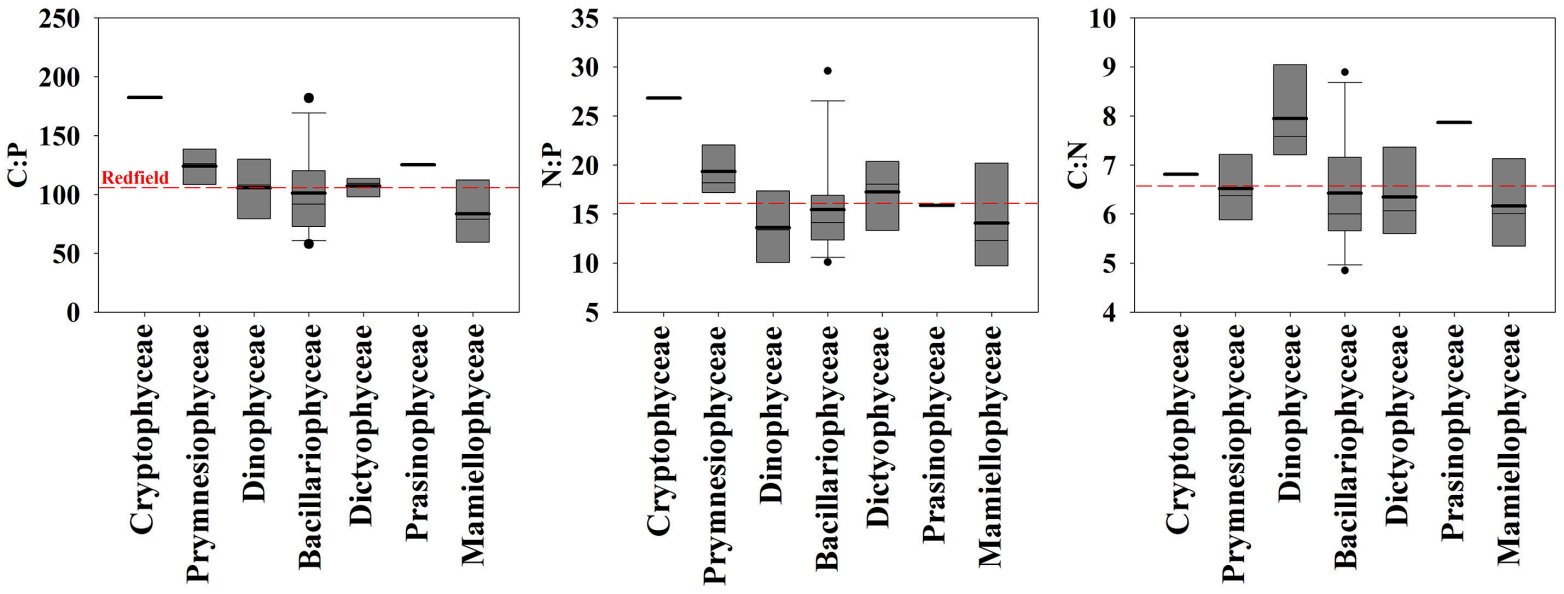
Comparison of cellular molar elemental ratios of carbon, nitrogen, and phosphorus among classes of 30 isolates of eukaryotic phytoplankton with box plots. The mean (bold line) and median (thin line) are plotted within the box which indicates quartiles. Whiskers indicate 10 and 90% quantiles and dots are outliers.

The mean growth rate relative to the mean maximum observed growth rate (μ:μ_max_; from triplicate cultures) was highly variable between isolates at the time of sampling (Table 1). For example, this ratio was below 0.10 for 3 of the 30 isolates, suggesting that the physiology of some of the isolates was in poor condition. The mean particulate organic nitrogen and phosphorus concentrations in cultures were well below the concentrations of nitrate and phosphate in the L1 medium (882 μM nitrate and 36 μM phosphate, see Table 1), indicating that cultures were not limited by these nutrients. Mean C:P, N:P, and C:N of all isolates were 107.3 ± 31.9 (s.d.), 16.2 ± 5.0 (s.d.), and 6.7 ± 1.1 (s.d.), respectively, reflecting ratios proposed by Redfield (1958) (Figure 1). Among specific isolates, we observed the highest mean C:P and N:P within the classes Cryptophyceae [*Chroomonas mesostigmatica*, (CCMP1168); C:P = 182.4 ± 3.9 s.d., N:P = 26.8 ± 1.2 s.d.] and Bacillariophyceae [*Thalassiosira rotula* (CCMP1018); C:P = 181.8 ± 54.2 s.d., N:P = 29.6 ± 1.1 s.d.] and the lowest within the class Mamiellophyceae [*Micromonas pusilla* (CCMP1723); C:P = 56.9 ± 9.0 s.d., N:P = 8.9 ± 1.2 s.d.] (Table 1). Mean C:N among isolates was highest within the class Dinophyceae [*Prorocentrum mexicanum* (CCMP687) C:N = 9.4 ± 0.3 s.d.] and lowest within the class Bacillariophyceae [*Thalassiosira oceanica* (CCMP1005); C:N = 4.8 ± 0.2 s.d.]. We observed the highest variability in C:P, N:P, and C:N within Bacillariophyceae (Figures 2, 3). We measured the largest cells within Dinophyceae [the isolate of *Karenia brevis* (CCMP687) had the largest mean cell volume, 13.1 × 10^3^ μm^−3^] and the mean cell volume within *Ostreococcus lucimarinus* (CCMP2972; Mamiellophyceae) was smallest (5.6 × 10^−3^ μm^−3^). Despite the strong contrast in cell size, mean growth rates, under these conditions, were lowest and nearly identical in Dinophyceae (0.08 ± 0.07 d^−1^ s.d.) and Mamiellophyceae (0.08 ± 0.1 d^−1^ s.d.) and highest in Bacillariophyceae (0.38 ± 0.30 d^−1^ s.d.) and Prymnesiophyceae (0.38 ± 0.29 d^−1^ s.d.).

To determine how phylogenetic structure was related to cellular elemental stoichiometry of phytoplankton, we compared the phylogenetic relationship of the 18S rRNA to the ratios of elements within cells. To broadly examine this relationship, we compared matrices of the 18S rRNA sequences (dissimilarity distance matrix) and stoichiometric ratios (Euclidean distance matrix) using the Mantel test (Mantel, 1967). The Mantel correlation was low and not significant for C:P (0.11, *p* = 0.10), N:P (0.11, *p* = 0.10), and C:N = (0.11, p = 0.08), indicating no relationship between phylogeny of the 18S rRNA sequence and cellular C:N:P stoichiometry within our 30 isolates.

**Figure 4:**
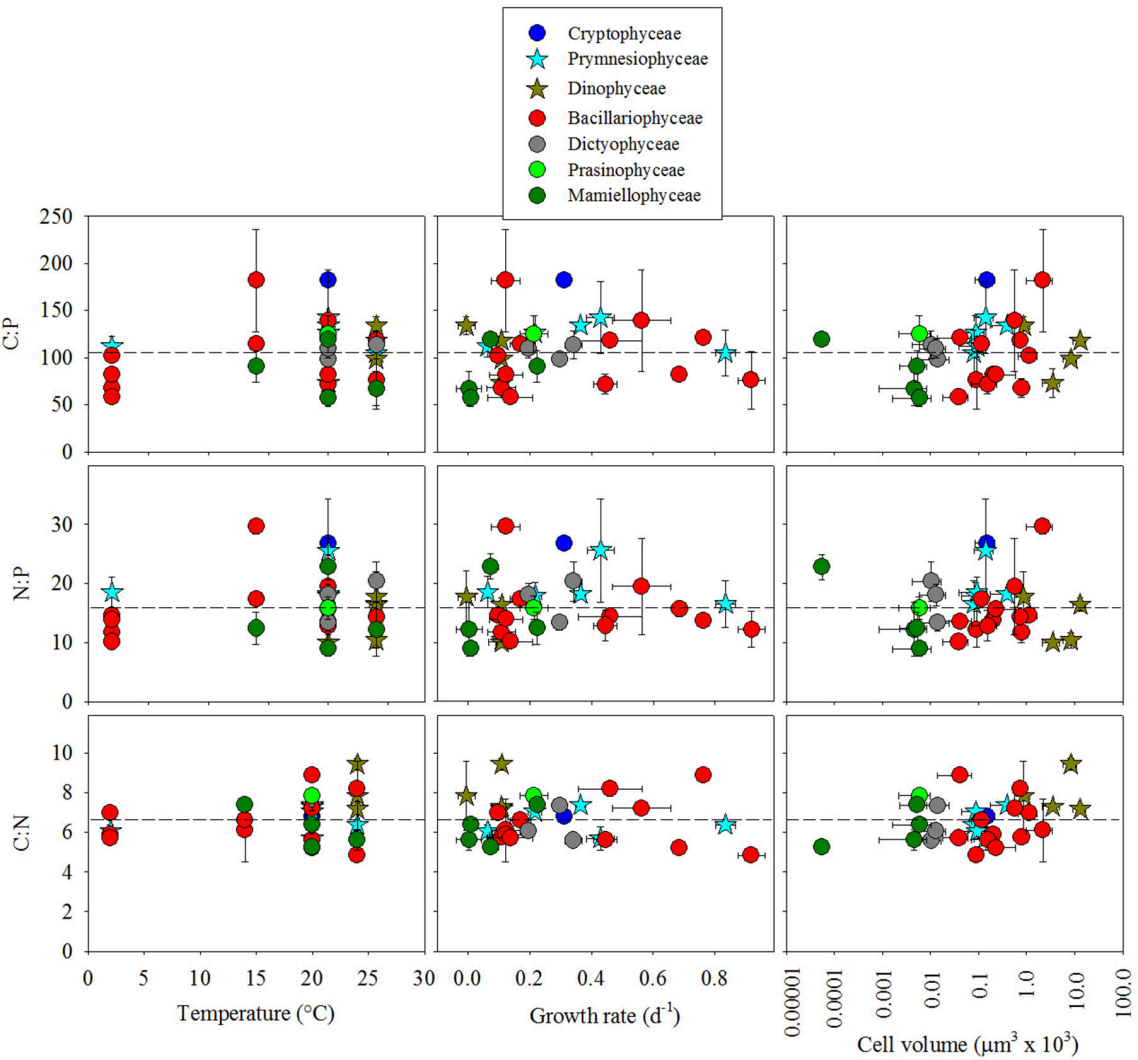
Cellular molar elemental ratios of carbon, nitrogen, and phosphorus among classes of 30 isolates of eukaryotic phytoplankton as a function of temperature, growth rate, and cell volume. Different colors separate classes and dotted lines indicate reference to Redfield ratios.

To determine how phylogenetic class, cell volume, growth rate, and temperature contribute to cellular CNP ratios, we fitted a general linear model (glm) to our data with the form *f* (x) = (x) (class + cell volume + growth rate + temperature) + ε. In general, stoichiometric variability of C:P, N:P, and C:N within classes was high (Figures 2, 3). Residuals form our statistical model were responsible for over half of the model variance for C:P (51.5%), N:P (57.3%), and C:N (70.4%) (ANOVA test on the glm). Phylogenetic class was identified as a significant contributor to the overall variance of C:P (43.3%, *p* < 0.05), but not on N:P (39.4, *p* > 0.05) or C:N (13.2%, *p* > 0.05). Tukey's analysis of means, however, did not identify significant differences in cellular C:N:P ratios between classes except for a difference in C:P between Mamiellophyceae (*n* = 4 isolates) and Cryptophyceae (*n* = 1 isolate). Although the glm indicated that temperature had a significant effect on C:P (*p* < 0.05; Figure 4), temperature only explained 5.1% of the statistical model variance, suggesting that temperature had a minor effect. Growth rate and cell volume were not significant predictors of C:P, N:P, or C:N (*p* > 0.05).

We also used the phylogenetic least squares (pgls) statistical model to constrain phylogenetic structure of the isolates (using the 18S rRNA sequence) and determine how cell volume, growth rate and temperature might interact with the phylogenetic relationship of the 18S rRNA sequence to influence cellular CNP stoichiometry in the 30 isolates. The pgls model (*f* (x) = x(cell volume + growth rate + temperature)) however, did not identify significant trends between cellular elemental ratios and any of these factors (*p* > 0.05; Figure 4).

**Figure 5:**
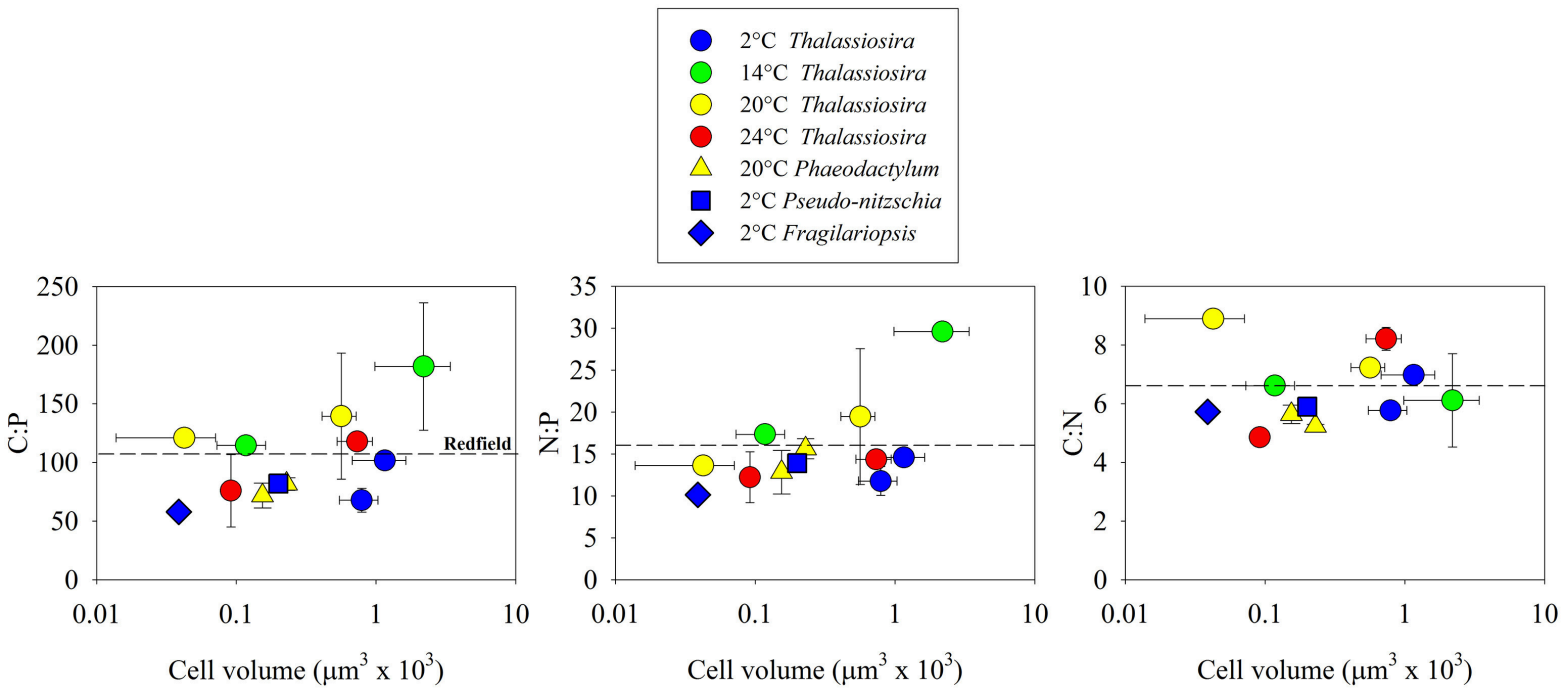
Cellular molar elemental ratios of carbon, nitrogen, and phosphorus within diatoms (Bacillariophyceae) as a function of cell volume, temperature, and genus. Dotted lines indicate reference to Redfield ratios.

We selected phytoplankton isolates to include a wide range in cell volume within Bacillariophyceae. Within this class, we also selected isolates that were collected from environments that have a wide range in temperature. Thus, 12 of the 30 isolates that we examined were diatoms, representing 40% of our analysis. Within Bacillariophyceae, mean C:P, N:P and C:N were close to Redfield values (101.1 ± 35.8 s.d.; 15.4 ± 5.1 s.d.; 6.3 ± 1.4 s.d., respectively; Figure 5). The glm (f(x) = (x) (cell volume + growth rate + temperature) + ε) indicated that cell volume had a significant positive effect on C:P and N:P, (*p* < 0.05; Figure 5; the effect on C:N was not significant *p* > 0.05) and accounted for a significant portion of the statistical model variance (*p* < 0.05; 46.6 and 59.4%, respectively). The pgls model also indicated that the robust effect of cell volume was the only significant factor affecting C:P and N:P (*p* < 0.05) within Bacillariophyceae.

## Discussion

We observe high variability in cellular elemental stoichiometry (C:N:P) within classes of phytoplankton (Figure 3). Furthermore, the phylogenetic relationship of the 18S rRNA sequence is not correlated with C:P, N:P, or C:N within our 30 isolates of eukaryotic phytoplankton, reflecting the absence of a relationship between phylogeny and elemental stoichiometry. Although we did not implement stringent physiological controls in our analysis, the effects of growth rate (i.e., μ/μ_max_) on elemental stoichiometry may be important in identifying relationships between phylogeny and elemental stoichiometry as identified previously (Quigg et al., 2003). We note, however, that environmental populations grow at variable rates. Thus, broad physiological ranges may be more important in identifying class-specific variability in elemental stoichiometry in the natural environment.

In comparison with other studies, our results corroborate recent findings but conflict with some current hypotheses. In our study, C:P and N:P variation among isolates within Bacillariophyceae is as large as the total variation of elemental ratios among all of the isolates that we examined (Figures 2, 3). This result is similar to findings from Finkel et al. (2016) where the species level was the largest source of variation in their hierarchical Bayesian analysis of macromolecular composition among lineages of phytoplankton. Our results also partially support previous studies that identify low C:P and N:P in cold-water diatoms (Arrigo et al., 1999, 2002). However, by including warm water diatom isolates, our data conflict with the hypothesis that all taxa within Bacillariophyceae have low C:P and N:P relative to other classes (Ho et al., 2003; Quigg et al., 2003). Thus, low cellular elemental ratios within diatoms may be common in cold-water isolates but elemental ratios were not consistently low in all of the diatom isolates that we examined and did not scale linearly with temperature (Figure 4). Our analysis, however, could be improved by including more isolates to account for larger variability within groups outside of Bacillariophyceae.

Our data also do not align with the general trend associated with the *growth rate hypothesis* (Sterner and Elser, 2002), whereby C:P and N:P ratios are expected to decline with increasing growth because of the high growth dependency on ribosomal P. Although several studies indicate that growth rate can have a strong relationship with C:P and N:P, there are many issues associated with this hypothesis in microalgae (Flynn et al., 2010) and may be restricted to phosphate-limited growth as identified previously (Goldman et al., 1979; Klausmeier et al., 2008; Garcia et al., 2016). This hypothetical change in C:P and N:P, for example, could be masked by cellular P storage in environments with a high P supply, as modeled by Klausmeier et al. (2004) but more generally, the growth-dependent change in the cellular ribosomal protein concentration seems to be a small fraction of a larger protein pool that changes as a function of microbial growth (Barenholz et al., 2016). The N and P input concentrations in our growth medium (L1) were considerably higher than the particulate concentrations of N and P in our cultures, indicating that growth rates were not limited by nutrients. Thus, the growth rate hypothesis may find more support in some natural environments where P is depleted relative to N.

Another related hypothesis suggests that temperature affects cellular elemental stoichiometry by modulating ribosome efficiency and hence the demand for ribosomal cellular P (Toseland et al., 2013). Their data suggest that cells compensate for low ribosomal efficiency at low temperatures by increasing the cellular concentration of ribosomes and hence cellular P (Toseland et al., 2013). Although our statistical model (glm) identified a significant effect of temperature on C:P and N:P, temperature only accounted for a small portion of the statistical model variance (5%). Thus, the effect of temperature on C:N:P variability within isolates might be more important than the effect on whole communities and may be more relevant for low P environments where P-storage does not interfere with the underlying effect on P-rich ribosome concentrations. With regard to the temperature-dependent translation efficiency hypothesis, the specific question about how temperature affects cellular elemental stoichiometry might be more thoroughly investigated with a full-factorial experimental design focusing on temperature with account for broad physiological effects like P-limited growth. Such an experimental design might consider an observable maximum growth rate and account for covariable relationships between temperature and growth rate (Boyd et al., 2013) and cell size and growth rate (Marañón et al., 2013). In addition to these considerations, however, adaptive mechanisms (e.g., such as those identified by Toseland et al., 2013) may further complicate relationships between cellular elemental stoichiometry, temperature, cell size and growth rate.

Although silicate concentrations in general, can be high in glass culturing flasks (50–100 μM.—M. Lomas unpublished data) we did not control for possible effects of silicate addition on C:N:P ratios. Such additional environmental factors may contribute to the variation in observed C:N:P ratios in microalgae including diatoms. The supply of silicate to diatoms, for example, may influence the proportion of cell volume that is occupied by other elements (Raven and Waite, 2004).

### Cell volume and C:N:P within diatoms

A large portion (40%) of our analysis focused on Bacillariophyceae to identify how cell size, growth rate, and temperature might contribute to C:N:P variability as diatoms represent a large portion of marine net primary production (Nelson et al., 1995; Armbrust et al., 2002). Of these 3 factors, our analysis identified a significant positive relationship between cell volume and C:P and N:P (Figure 5). Because of the limited number of isolates that we analyzed in other classes of phytoplankton, we cannot directly compare this trend in Bacillariophyceae with other classes but one feature that is unique to Bacillariophyceae is the low carbon and nitrogen investments in the protective layer surrounding cells, as this group depends on silica for major support in addition to the silicolemma. This is in contrast to other phytoplankton lineages, like chlorophytes that have high concentrations of glycoproteins in the cell wall (Northcote and Goulding, 1958; Gerken et al., 2013). Another factor that might contribute to a large portion of C:N:P variability in some diatoms is β-chitin. Of the 12 isolates that we examined, 8 belong to the genus *Thalassiosira*, which is known to invest C and N at a ratio of 8:1 in β-chitin spines that extend through the silica shell to reduce sinking rates (McLachlan et al., 1965; McLachlan and Craigie, 1966; Round et al., 1990; Durkin et al., 2009). Thus, the positive relationship between cell volume and C:N:P within Bacillariophyceae could result from a combination of silica-based cell structure and C:N-enriched β-chitin, specifically within *Thalassiosira*. Although not unique to Bacillariophyceae, lipids are an important carbon storage mechanism (McGinnis et al., 1997), which could also contribute to the positive relationship between C:P and cell volume. This positive relationship within Bacillariophyceae is in contrast to other data supporting the hypothesis that isolates with small cells have high C:P and N:P (Bertilsson et al., 2003; Martiny et al., 2013) and should be investigated further because of the projected surface-ocean warming and its associated effect of declining nutrient concentrations on phytoplankton cell size (Finkel et al., 2005, 2007, 2010). Further investigations might identify more complex effects of vacuoles on this relationship.

In summary, we identified a high degree of variability in C:P and N:P and C:N within the class Bacillariophyceae that is as large as the stoichiometric variability between the 7 classes that we examined. While some of our data support previous studies in this regard, this high variability is in contrast to several studies that identify only low ratios of C:P and N:P in diatoms in comparison with other lineages. Whereas, previous studies focus on a single factor relationship between phylogeny and cellular elemental stoichiometry, our analysis includes effects from multiple variables that are currently thought to affect cellular elemental stoichiometry. Overall, our study highlights the complexity of variability in cellular elemental ratios among marine phytoplankton. Generally, our results suggest that the link between changes in ocean phytoplankton community composition and C:N:P is complex and we cannot simply assume that the presence of diatoms leads to low ratios in ocean regions. Our study further identifies the need to control for physiological effects to test current hypotheses relating to potential trends in phytoplankton elemental stoichiometry. Future studies that use different sources of nutrients (e.g., ammonium vs. nitrate) might improve our understanding of variability in physiology and elemental stoichiometry between phytoplankton groups.

## Author contributions

NG and AM contributed to the experimental design, statistical analyses, and writing. ML contributed to the experimental design, sample collection and writing. JS, JB, and TR contributed to the experimental design, culturing phytoplankton, and sample collection.

### Conflict of interest statement

The authors declare that the research was conducted in the absence of any commercial or financial relationships that could be construed as a potential conflict of interest.

## Supplementary material

Figure S1 Relative fluorescence units of *in vivo* Chl *a* in 30 isolates of marine eukaryotic phytoplankton. Symbols are color-coded by class: dark yellow, Dinophyceae; dark green, Mamiellophyceae; dark blue, Cryptophyceae; light blue, Prymnesiophyceae; gray, Dictyophyceae; light green, Prasinophyceae. Species names are color-coded by temperature. Growth rates were calculated between the 2-day period before terminal sampling of cultures.

Figure S2 Observed growth rates in 30 isolates of marine eukaryotic phytoplankton. Symbols are color-coded by class: dark yellow, Dinophyceae; dark green, Mamiellophyceae; dark blue, Cryptophyceae; light blue, Prymnesiophyceae; gray, Dictyophyceae; light green, Prasinophyceae. Species names are color-coded by temperature. Growth rates were calculated between the 2-day period before terminal sampling of cultures. Black numbers in the lower left of the panel are cell volumes estimates in μm^3^ × 10^3^ (estimates correspond with data in Figures 4, 5).
