# Supplementary figures and images for: High Variability in Cellular Stoichiometry of Carbon, Nitrogen, and Phosphorus Within Classes of Marine Eukaryotic Phytoplankton Under Sufficient Nutrient Conditions
Source: Front Microbiol. 2018 Mar 27;9:543. doi: 10.3389/fmicb.2018.00543 (PMC5880891; doi:10.3389/fmicb.2018.00543)

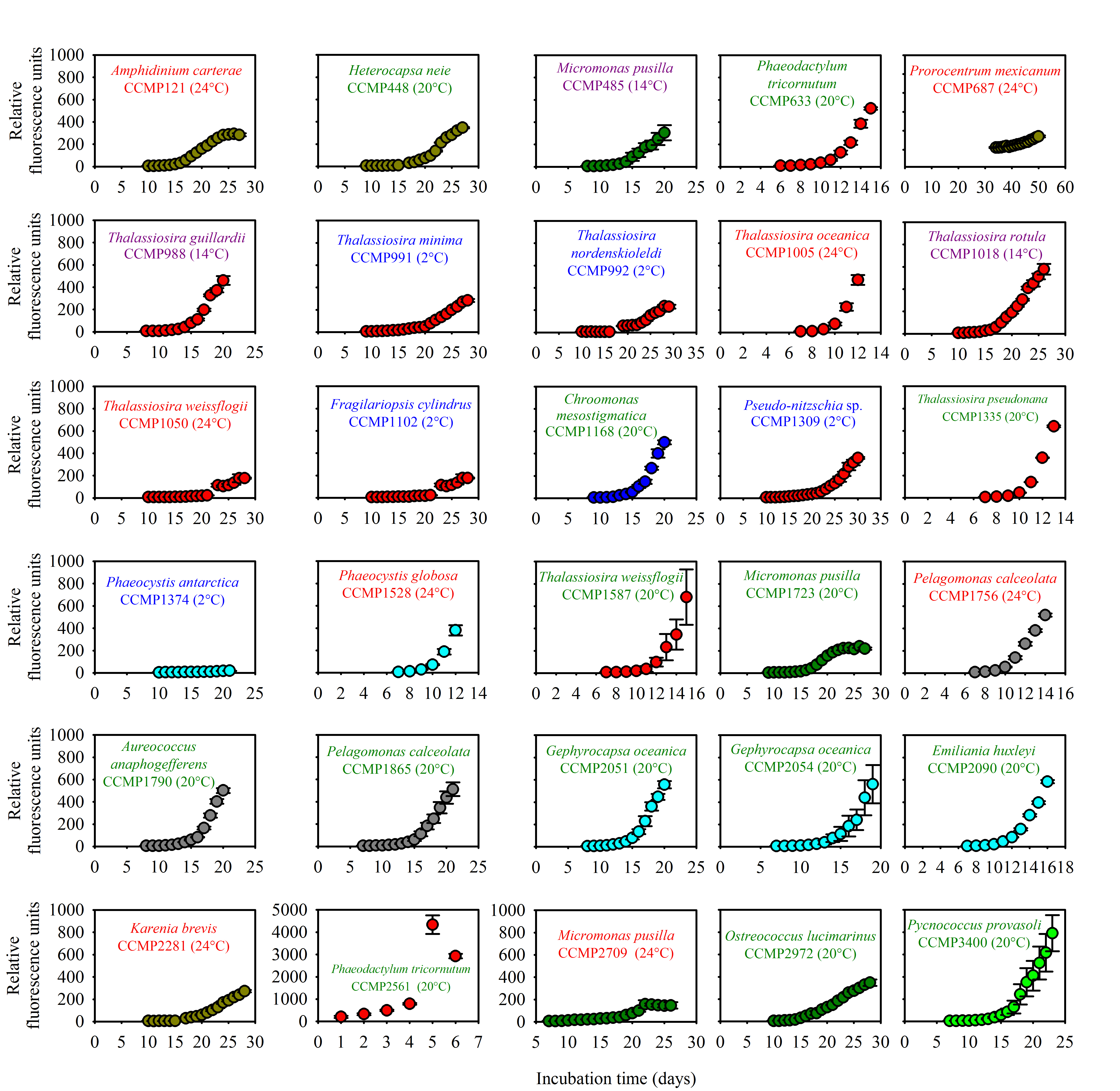

Supplement: Figure S1 — Relative fluorescence units of in vivo Chl a in 30 isolates of marine eukaryotic phytoplankton. Symbols are color-coded by class: dark yellow, Dinophyceae; dark green, Mamiellophyceae; dark blue, Cryptophyceae; light blue, Prymnesiophyceae; gray, Dictyophyceae; light green, Prasinophyceae. Species names are color-coded by temperature. Growth rates were calculated between the 2-day period before terminal sampling of cultures. [file Image1.JPEG]

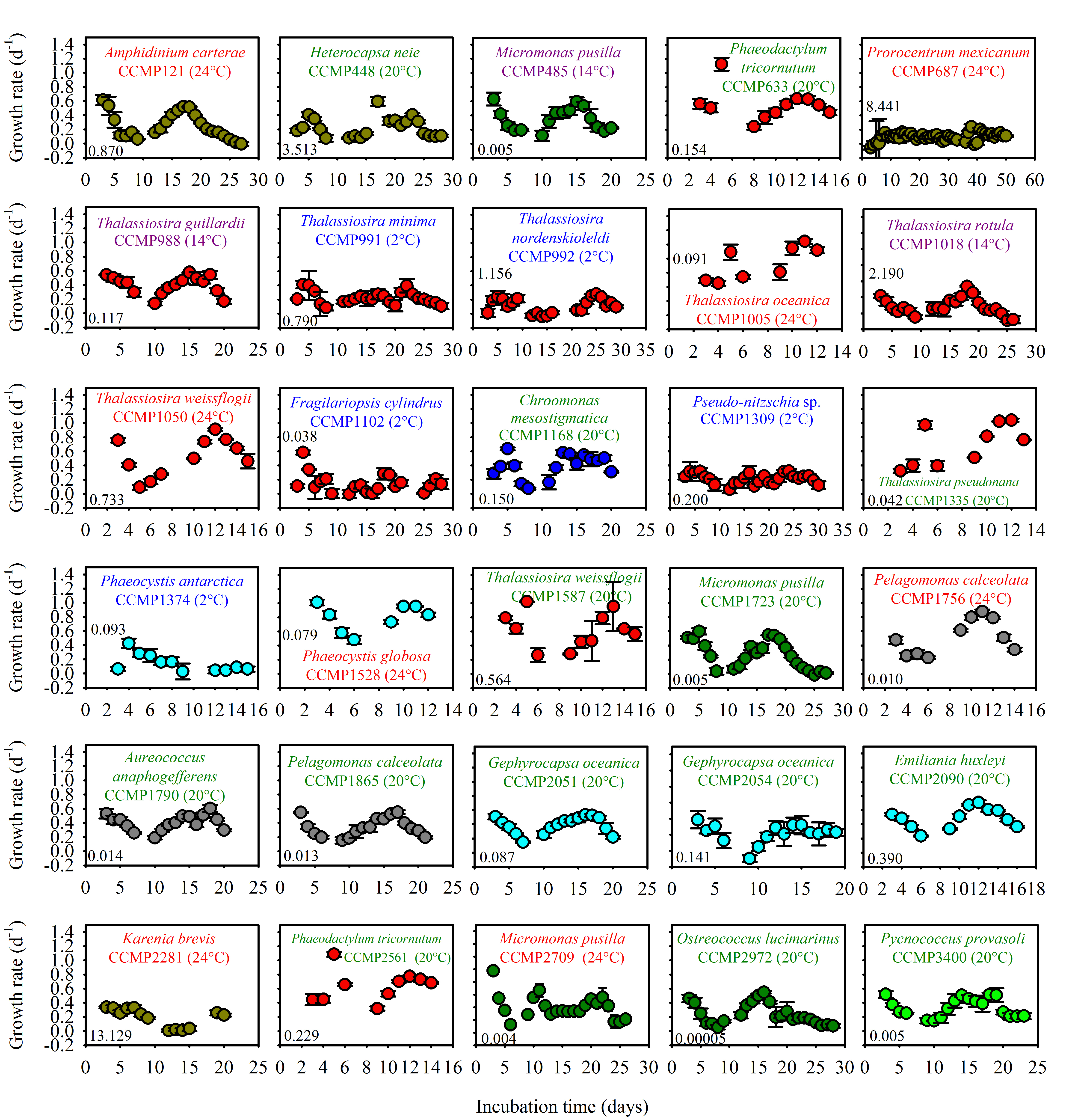

Supplement: Figure S2 — Observed growth rates in 30 isolates of marine eukaryotic phytoplankton. Symbols are color-coded by class: dark yellow, Dinophyceae; dark green, Mamiellophyceae; dark blue, Cryptophyceae; light blue, Prymnesiophyceae; gray, Dictyophyceae; light green, Prasinophyceae. Species names are color-coded by temperature. Growth rates were calculated between the 2-day period before terminal sampling of cultures. Black numbers in the lower left of the panel are cell volumes estimates in μm3 × 103 (estimates correspond with data in Figures 4, 5). [file Image2.JPEG]
